# LDL binding to cell receptors and extracellular matrix is proatherogenic in obesity but improves after bariatric surgery

**DOI:** 10.1016/j.jlr.2023.100451

**Published:** 2023-09-28

**Authors:** Shobini Jayaraman, Antonio Pérez, Inka Miñambres, Jose Luis Sánchez-Quesada, Olga Gursky

**Affiliations:** 1 Department of Pharmacology, Physiology & Biophysics, Chobanian and Avedisian School of Medicine, Boston University, Boston, MA, USA; 2 Endocrinology Department, Hospital de la Santa Creu i Sant Pau, Barcelona, Spain; 3 CIBER of Diabetes and Metabolic Diseases (CIBERDEM), Barcelona, Spain; 4 Cardiovascular Biochemistry Group, Research Institute of the Hospital de Sant Pau, CIBERDEM, Barcelona, Spain

**Keywords:** LDL binding to LDL receptor, CD36 and LOX-1, LDL retention by matrix proteins and glycosaminoglycans, Inflammation in obesity, Lipoprotein lipolysis, oxidation and aggregation

## Abstract

Obesity is a major global public health issue involving dyslipidemia, oxidative stress, inflammation, and increased risk of CVD. Weight loss reduces this risk, but the biochemical underpinnings are unclear. We explored how obesity and weight loss after bariatric surgery influence LDL interactions that trigger proatherogenic versus antiatherogenic processes. LDL was isolated from plasma of six patients with severe obesity before (basal) and 6–12 months after bariatric surgery (basal BMI = 42.7 kg/m^2^; 6-months and 12-months postoperative BMI = 34.1 and 30 kg/m^2^). Control LDL were from six healthy subjects (BMI = 22.6 kg/m^2^). LDL binding was quantified by ELISA; LDL size and charge were assessed by chromatography; LDL biochemical composition was determined. Compared to controls, basal LDL showed decreased nonatherogenic binding to LDL receptor, which improved postoperatively. Conversely, basal LDL showed increased binding to scavenger receptors LOX1 and CD36 and to glycosaminoglycans, fibronectin and collagen, which is proatherogenic. One year postoperatively, this binding decreased but remained elevated, consistent with elevated lipid peroxidation. Serum amyloid A and nonesterified fatty acids were elevated in basal and postoperative LDL, indicating obesity-associated inflammation. Aggregated and electronegative LDL remained elevated, suggesting proatherogenic processes. These results suggest that obesity-induced inflammation contributes to harmful LDL alterations that probably increase the risk of CVD. We conclude that in obesity, LDL interactions with cell receptors and extracellular matrix shift in a proatherogenic manner but are partially reversed upon postoperative weight loss. These results help explain why the risk of CVD increases in obesity but decreases upon weight loss.

Obesity is a growing public health problem worldwide that currently affects over 40% adults in the United States of America; alarmingly, over 9% of the USA adults had severe obesity in 2020, and the numbers keep growing [https://www.cdc.gov/obesity/data/adult.html], providing a major risk factor for CVD, diabetes, and certain cancers. Obesity often involves hyperlipidemia, diabetes, hypertension, chronic inflammation, and oxidative stress (1, 2, 3), which increase susceptibility to CVD. This increased susceptibility is generally attributed to the proatherogenic changes in plasma lipoproteins, including elevated triglycerides, mainly in the form of VLDL, as well as decreased levels and impaired functionality of HDL (4, 5), including smaller HDL particles with diminished antioxidant capacity (6). Notably, LDL are the major risk factor for CVD, yet no changes in LDL levels associated with obesity or weight loss have been reported to date, and surprisingly little is known about LDL composition or functionality in obesity (7). The present study addresses this gap in knowledge by exploring composition and functionality of single-donor LDL from plasma of subjects with severe obesity before and after bariatric surgery.

If nonsurgical approaches to weight loss fail, surgical treatments can be effective for severe obesity. Bariatric surgery often leads to rapid, sustained weight loss, lowers CVD mortality (8), and improves lipoprotein levels, including decreased triglycerides and increased HDL cholesterol (4, 5). HDL quality also improves, including a shift from smaller to larger particles (9, 10) and increased capacity to promote cellular cholesterol efflux (11). Little is known about postoperative changes in other lipoproteins. Our team previously explored biochemical properties and susceptibility to enzymatically induced aggregation of LDL and oxidation of LDL and HDL from 13 patients with severe obesity before and up to one year after bariatric surgery (7). While biochemical composition of HDL and VLDL was altered in obesity and was partially normalized after surgery, no changes in LDL composition were detected. Notably, increased LDL propensity to sphingomyelinase-induced aggregation, copper-induced oxidation, and increased population of electronegative LDL, termed LDL(−), was observed, suggesting proapoptotic and proatherogenic processes (7, 12). To understand molecular underpinnings of these processes, the present study explored biochemical and functional properties of LDL from a subset of these patients.

LDL functionality depends on the nonatherogenic lipoprotein uptake by peripheral cells via LDL receptor (LDLR) versus proatherogenic uptake by arterial macrophages via scavenger receptors, leading to foam cell formation. LDL–LDLR interactions involve specific binding of basic residue arrays in apoB to the acidic ligand–binding regions in LDLR (13). Oxidation of LDL diminishes its affinity for LDLR (14, 15), reflecting oxidative lysine modifications in the LDLR-binding site of apoB (16). Conversely, LDL binding to scavenger receptors such as CD36 or LOX-1 increases upon LDL oxidation as it is driven by nonspecific receptor binding to oxidized lipids (17). Contributing to this proatherogenic pathway is LDL retention in the arterial wall matrix, a critical step in the initiation and progression of atherosclerosis (18). LDL binds via its major protein, apoB, and minor proteins, such as lipoprotein(a), serum amyloid A (SAA), and apoE, to smooth muscle cell-derived extracellular proteoglycans, including glycosaminoglycans (GAGs) (19, 20). LDL oxidation, lipolysis, glycation, and aggregation can augment LDL binding to proteoglycans (21, 22). Conversely, proteoglycan binding promotes proatherogenic LDL modifications in the arterial wall, such as oxidation and fusion, thereby perpetuating the vicious circle (23).

Two LDL subclasses, LDL(−) and small dense LDL, are associated with obesity, type-2 diabetes, and inflammation (12, 24). These subclasses are believed to be particularly proatherogenic for several reasons. First, their decreased affinity for LDLR prolongs their residence time in the bloodstream, increasing the probability of proatherogenic modifications. Second, they penetrate the arterial wall more easily, bind more strongly to arterial proteoglycans, and hence are retained more readily. Third, they are more prone to fusion, which enhances lipoprotein retention in the arterial wall (25, 26). Finally, they are more susceptible to oxidation, which drives LDL uptake via scavenger receptors (24).

Furthermore, LDL vary in the composition of minor proteins, some of which provide additional cardiovascular risk factors. These include: lipoprotein (a), Lp(a), a prothrombotic and proatherogenic LDL-like particle that contains lipoprotein(a) moiety covalently bound to apoB (27); lipoprotein-associated phospholipase A_2_ (Lp-PLA_2_) that preferentially hydrolyses oxidized phospholipids, which promotes atherosclerotic plaque buildup (28); and SAA, an inflammatory marker that in obesity preferentially associates with LDL (7, 29, 30). Lp(a) and SAA enhance lipoprotein binding to proteoglycans ((27, 31) and references therein), while NEFA generated by Lp-PLA_2_ and other lipases promote proatherogenic LDL fusion (20, 31). Measurement of LDL subclasses with enhanced proatherogenic properties such as these can provide new insights into the links between obesity and CVD.

In the current study, we examined the impact of obesity and bariatric surgery on LDL properties that are expected to contribute to the development of atherosclerosis, including the levels of NEFA, SAA, and Lp(a), as well as the population of endogenous aggregated/fused LDL and LDL(−). We explored LDL binding to cell receptors LDLR, CD36, and LOX-1, and to several extracellular matrix components. To our knowledge, such multicomponent binding studies of LDL functionality have not been previously reported. The results reveal that LDL functionality is altered in obesity in a proatherogenic manner but is partially restored upon postoperative weight loss. These findings help establish molecular basis for the causal link between obesity-induced inflammation and increased risk of CVD. They also help better understand the benefits and limitations of bariatric surgery in normalizing lipoprotein metabolism and cardiovascular health.

## Materials and methods

### Patients’ selection

Plasma LDL from six patients with obesity and six healthy normolipidemic controls were explored. This was a subset of 13 patients with obesity and 13 controls explored in the previous study that describes the criteria for patients’ selection and other details (7). The study was performed in accordance with the Helsinki Declaration. All subjects gave written informed consent before participating in the study, and the protocol was approved by the ethical committee of the Hospital de la Santa Creu i Sant Pau. Briefly, the patients had severe obesity and underwent bariatric surgery at Hospital de Sant Pau, Barcelona, Spain. All patients had BMI >40 kg/m^2^ or BMI >35 kg/m^2^ with comorbidities, including hypertension, diabetes, or hyperlipidemia. None of the patients had inflammatory or infectious diseases and none was receiving anti-obesity or anti-inflammatory drugs. The patients underwent sleeve gastrectomy or gastric bypass surgery.

Six patients were selected for the current substudy based on the volume of the available plasma that was necessary to obtain sufficient amounts of LDL to complete the experiments. Average BMI of patients included in this substudy was 43.7 ± 4.1 kg/m^2^ (basal, preoperative), 33.8 ± 2.4 kg/m^2^ (6 months postoperatively), 29.9 ± 3.5 kg/m^2^ (one year postoperatively), and 24.1 ± 2.4 kg/m^2^ (controls). These and other patients’ parameters, their levels of selected proteins and lipids in plasma, and the levels of lipoprotein cholesterol are listed in supplemental Table S1. None of the patients received lipid-lowering treatment. Two patients presented with mild diabetes (HbA1c of 6.6% and 6.8%) and were treated with metformin before surgery; the treatment continued after surgery, suggesting that changes in LDL observed postoperatively were due to surgery rather than metformin treatment.

### LDL preparation

Blood samples were obtained one week before the surgery (basal) and at 6 and 12 months thereafter. Blood was collected in EDTA-containing Vacutainer tubes after an overnight fast of 12 h. Plasma was obtained by centrifugation at 1,500 *g* at 4°C for 15 min. Plasma samples were frozen and stored at −80°C until use. LDL was isolated from plasma by KBr density ultracentrifugation (density range 1.019–1.063 g/ml); the aliquots were stored in 10% sucrose at −80°C for up to 1 year until use. Prior to experiments, the aliquots were thawed, LDL samples were dialyzed against PBS to remove sucrose, stored at +4°C, and used as soon as possible in less than one month to minimize potential modifications. To verify that LDL isolated by this method was not contaminated with VLDL remnants, additional centrifugation was performed for two different control LDL and two basal LDL (four samples in total). LDL samples (100 μl of 0.5 mg/ml LDL protein adjusted to a total volume of 5 ml with KBr solution) were subjected to density gradient centrifugation in the range of 1.006–1.063 g/ml. Lipoproteins were collected in 500 μl fractions. Essentially all lipoproteins were recovered in the density range of 1.019–1.060 g/ml, well above the density of VLDL (0.83–1.006 g/ml) and remnants (1.006–1.019 g/ml). Therefore, LDL in the current study was free from VLDL and remnants.

### Protein and lipid quantification

Total protein concentration was determined using a Quick Start Bradford protein assay kit (Bio-Rad, #5000201) with BSA as a standard. Individual protein levels were measured using commercially available ELISA kits from Abcam: human apoB (#AB190806), human apoE (#AB233623), human Lp(a) (#AB212165), and human SAA (#AB100635). Lipids were quantified using commercial kits for cholesterol (Sigma, #MAK-043), triglycerides (Thermo Fisher Scientific, #ETGA-200), phospholipids (Thermo Fisher Scientific #EPLP-100) and NEFA (Thermo Fisher Scientific, #EFFA-100). All concentrations were measured in technical triplicates.

### Thiobarbituric acid reactive substances measurements

The endogenous levels of peroxidized lipids in LDL were assessed by thiobarbituric acid reactive substance (TBARS) formation (32). Briefly, samples were incubated with 0.5 ml of 20% acetic acid (pH 3.5) and 0.5 ml of 0.78% aqueous solution of thiobarbituric acid (Sigma, #T5500). After incubating at 95°C for 45 min, the samples were centrifuged at 4000 rpm for 5 min. The red pigment in the supernatant fractions was estimated by absorbance at 532 nm. A calibration curve was generated using a malondialdehyde standard (Sigma, #36357). Results were expressed as μmol malondialdehyde per mg of LDL protein. The results for all samples were within the linear portion of the standard curve.

### Liquid chromatography

Liquid chromatography was performed on ÄKTA UPC 10 fast protein liquid chromatography system (GE Healthcare). A Superose 6 10/300 GL column was used for size-exclusion chromatography. LDL was eluted with 10 mM PBS, pH 7.4 at a flow rate of 0.5 ml/min. The HiTrap-HP column (GE Life Sciences, #17040601) was used for heparin affinity chromatography (21). The column was equilibrated with five volumes of buffer A (10 mM sodium phosphate, pH 7.4). The LDL solution (0.5–0.8 mg protein) was filtered with a 0.2 μm filter and applied to the column. The flow-through fraction was eluted with three column volumes of buffer A, and the bound fraction was eluted with three volumes of buffer B (10 mM sodium phosphate buffer, 1 M NaCl, pH 7.4). Anion exchange chromatography was performed using HiTrap Capto Q (Cytiva, #17547051); LDL samples were dialyzed against 10 mM Tris, 1 mM EDTA, pH 7.4, and were eluted with a stepwise salt gradient of 0.0–1.0 M NaCl as previously described (33).

### LDL binding to matrix components measured by ELISA

LDL binding to the extracellular matrix components was measured by ELISA following published protocols (34, 35) with slight modifications (36). Polystyrene 96-well plates (Nunc Immunosorp plates, Sigma #Z755273) were incubated overnight at 4°C either with 5 mg/ml of BSA (Sigma, #A3294) or with 100 μl of 0.1 mg/ml matrix glycans and/or proteins, including: heparin (Sigma, #H4784), heparan sulfate (Sigma, #H7640), chondroitin sulfate (Sigma, #C9819), hyaluronic acid sodium salt (Sigma, #40583, 50 μg/ml in PBS, pH 7.4), collagen type-IV from human placenta (Sigma, #CC076), recombinant human biglycan protein (Bio-techne R&D, #2667-CM-050), fibronectin (Sigma, #11051407001), and recombinant human endorepellin perlecan protein (Bio-techne R&D, #2364-ER-050, 10 μg/ml in PBS, pH 7.4). After coating, the wells were blocked with 3% BSA, 1% fat-free milk powder, and 0.05% Tween-20 in PBS (PBST) for 1 h at 37°C. Wells coated with BSA alone were used as controls. The plates were incubated with LDL (5–25 μg protein) for 1 h at 37°C in a buffer containing 1% BSA, 10 mM Hepes, 150 mM NaCl, 2 mM CaCl_2_, 2 mM MgCl_2_ at pH 7.5. After incubation, the wells were washed thrice with a buffer containing 50 mM NaCl, 2 mM CaCl_2_, 2 mM MgCl_2_, 10 mM Hepes at pH 7.5. The amounts of bound lipoproteins were measured using an antibody for apoB. After three washes with PBST, the plates were incubated for 1 h with an anti-human apoB monoclonal antibody (Santa Cruz #SC-13538 HRP) diluted at 1:4000. The plates were washed with PBST, and a substrate solution of 3,3′,5,5′-tetramentylbenzidine (Thermo Fisher Scientific, #34028) was added to the plates. A colorimetric assay was performed at 450 nm using a microplate reader (Tecan infinite M1000).

Well coating with sulfated proteoglycans was verified using a colorimetric assay with 1,9-dimethylmethylene blue (DMMB) (37) (Sigma, #341088). DMMB stock solution (1.6 mg DMMB in 100 ml water) was filtered using 0.2 μm filter. The assay buffer (3.04 g glycine, 1.6 g NaCl in 95 ml acetic acid, pH 3) was freshly prepared, and 100 μl of buffer was added to each well followed by addition of 100 μl DMMB stock solution. In proteoglycan-coated well, the color changed immediately from blue to pink. Binding assays were validated using control LDL from lean subjects.

Single point measurements using LDL from patients with obesity before and after surgery were performed using 10 μg LDL protein per test. To determine specific binding of LDL to matrix components or cell receptors (described in the next section), the measurements were corrected for nonspecific binding to BSA-coated wells. The nonspecific binding accounted for <5% of the total binding to the receptor- or proteoglycan-coated wells. The assay was performed during several weeks. The day-to-day variations were below 15%; to account for it, a control LDL sample was analyzed in each plate; no drift in the assay was noted. The variation between duplicate measurements carried out in separate well plates was under 3%.

### LDL binding to cell receptors measured by ELISA

LDL binding to three cell receptors was measured by ELISA; this included recombinant human LDLR (Abclonal #RP00152), His-tagged extracellular ligand-binding domain of human CD36 (Sino Biologicals Inc., #10752-1708H), and recombinant human LOX-1/OLR1 (Bio-techne R&D, #1798-LX-050). Receptors (10 μg/well, 100 μl in 10 mM PBS, pH 7.4) were immobilized on a 96-well plate (Nunc Immunosorp plates, Sigma, #Z755273) and incubated at 4°C overnight. Wells coated with BSA (5 mg/ml) were used as controls. After washing with 1× PBST, the plates were blocked with 3% BSA-PBST containing 2 mM EDTA for 1 h. After blocking, LDL (5–25 μg protein) in a buffer containing 1% BSA, 10 mM Hepes, 150 mM NaCl, 2 mM CaCl_2_, 2 mM MgCl_2_ at pH 7.5 was added, and the samples were incubated at 37°C for 1 h. Next, the wells were washed thrice with 1× PBST and incubated with anti-human apoB (1:4000 in BSA-PBST) for 1 h at room temperature. The plates were washed thrice with 1× PBST and a substrate solution (3,3′,5,5′-tetramentylbenzidine) was added to each well. A colorimetric assay was performed at 450 nm using a microplate reader (Tecan Infinite 1000). The assay for LDLR was validated using control intact LDL. Validation for CD36 and LOX-1 was performed using control LDL that were oxidized by copper. To this end, LDL (0.2 mg/ml protein) were incubated with 5 μM CuSO_4_ at 37°C for 12 h, the reaction was quenched by 1 mM EDTA, and LDL were dialyzed against 10 mM PBS, pH 7.4 prior to use.

### Isolation of human serum albumin from lipoprotein-deficient serum

Human serum albumin was purified from lipoprotein-deficient serum (d > 1.22 g/ml KBr) using HiTrap blue HP affinity column (#17041201, Cytiva). The column was equilibrated with five volumes of buffer A (20 mM sodium phosphate, pH 7.4). The lipoprotein-deficient serum (50 mg protein) was filtered with a 0.2 μm filter and applied to the column. The flow-through fraction was eluted with five column volumes of buffer A, and the bound fraction was eluted with 10 volumes of buffer B (20 mM sodium phosphate buffer, 1 M NaCl, pH 7.4). The albumin purity was confirmed by SDS PAGE and the concentration of purified albumin was determined by Bradford assay.

### LDL oxidation and enzymatic lipolysis

LDL oxidation by Cu^2+^ was carried out at 37°C for 12 h as previously described (21)). Briefly, LDL solutions containing 0.5 mg/ml protein were equilibrated at 37°C; the oxidation was initiated by adding 10 μM CuSO_4_. The extent of reaction was monitored by absorbance at 234 nm for conjugated diene formation.

For lipolysis, LDL was incubated for 6 h at 37°C in buffer (150 mM NaCl, 2 mM CaCl_2_, 20 mM Tris HCl, pH 7) with 10 μg/ml secretory PLA_2_. The lipolysis was stopped by adding EDTA to a final concentration of 10 mM. The degree of PLA_2_-induced lipolysis was determined by measuring the amounts of NEFA as previously described (21). Modified LDL were dialyzed against 10 mM PBS before use.

### Statistical analysis

All experiments were performed in five replicates. Data analysis was performed using GraphPad Prism 9.0, version 9.5.1 (San Diego, CA) and Origin, version 7.0383 (OriginLab Corporation, MA). The statistical significance of the difference between two measurement groups was determined using the nonparametric unpaired *t* test. Average values of five replicates are shown by data points, with median ± SD shown by bars; *P* <0.05 was considered significant.

## Results

### Plasma lipid profiles and inflammation markers in obesity before and after surgery

The clinical characteristics, plasma lipid profiles, and plasma inflammatory markers of our subgroup of patients with obesity at basal level, 6 months, and one year after bariatric surgery are listed in supplemental Table S1 and in (7). Briefly, the basal levels of total plasma cholesterol, LDL-cholesterol, and plasma apoB were similar to controls. Basal plasma triglycerides and VLDL-cholesterol were elevated, while basal HDL-cholesterol levels and antioxidant HDL capacity were decreased. One-year postsurgery, plasma triglycerides and VLDL-cholesterol decreased but remained slightly higher than controls, while HDL properties largely normalized. In particular, plasma triglycerides decreased from the basal level of 1.67 ± 0.92 mmol/l to 1.06 ± 0.30 mmol/l one year postoperatively, as compared to 0.73 ± 0.16 mmol/l in controls. Notably, NEFA plasma levels were comparable in all groups. C-reactive protein (CRP) was greatly elevated in basal plasma (10.5 ± 6.2 mg/l) versus controls (0.9 ± 0.3 mg/l), indicating systemic inflammation characteristic of severe obesity. Despite a major progressive postoperative decline, which is consistent with other studies (38, 39, 40, 41), plasma CRP remained elevated after surgery (3.8 ± 3.4 after 6 months, 3.3 ± 2.2 mg/l after one year).

### Biochemical composition of basal and postoperative LDL suggests obesity-associated inflammation

**Fig. 1:**
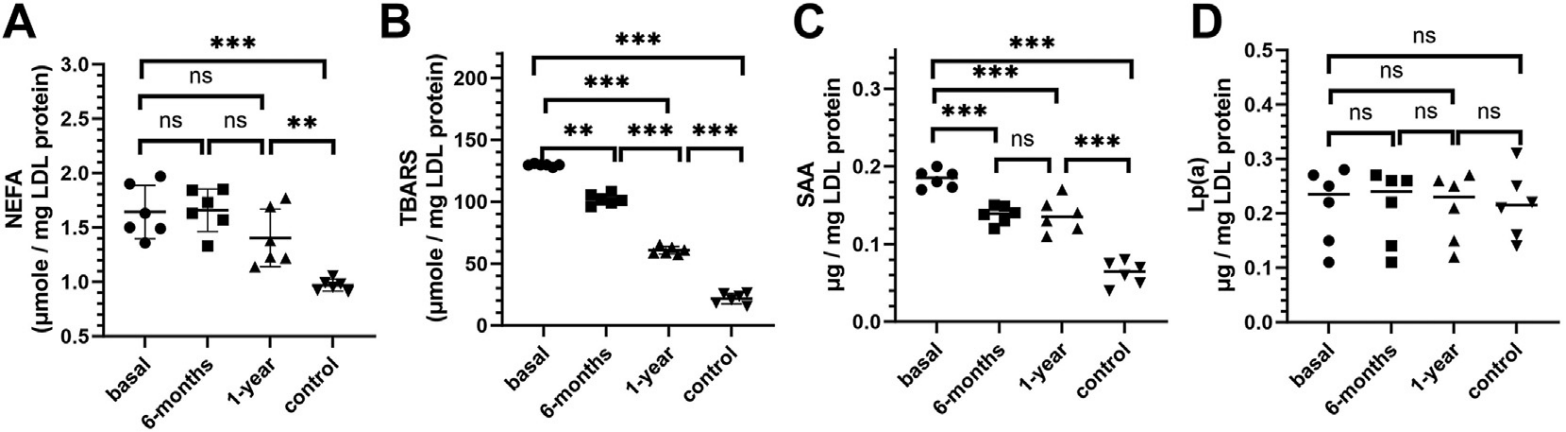
Inflammatory and proatherogenic markers in LDL from patients with obesity (basal, 6 months and one year after surgery) versus normolipidemic healthy controls. A: NEFA and (B) TBARS were measured using standard assays; (C) SAA and (D) Lp(a) were measured using ELISA kits. Each data point represents an average of five independent measurements. The bars show median ±SD; ns, not significant, ∗*P* < 0.05, ∗∗*P* < 0.01, and ∗∗∗*P* < 0.001. SAA, serum amyloid A; TBARS, thiobarbituric acid reactive substance.

First, we measured the major LDL lipids and proteins. No significant differences in the levels of triglycerides, cholesterol, phospholipids, total protein, apoB, or apoE were observed in basal versus control LDL (supplemental Fig. S1), consistent with prior study (7). Next, we measured endogenous inflammatory markers in LDL. NEFA content was elevated in basal versus control LDL and showed only marginal postoperative decline (Fig. 1A), suggesting that obesity promoted LDL lipolysis. Lipid peroxidation was greatly elevated at baseline and progressively declined 6- and 12-months postsurgery but remained higher than controls, as shown by TBARS assay (Fig. 1B). Therefore, obesity-associated lipolysis and lipid peroxidation were not completely resolved one-year postsurgery.

Furthermore, we measured the LDL-bound SAA, an inflammatory marker associated with LDL in obesity (7, 29, 30), and Lp(a), a particularly proatherogenic LDL-like lipoprotein that binds to extracellular matrix components ((27) and references therein). SAA content was increased in basal versus control LDL and, despite initial postoperative decline, remained elevated after one year (Fig. 1C), suggesting continued obesity-associated inflammation. In contrast, Lp(a) fraction of LDL, which varied widely among patients with obesity as well as controls, remained unchanged after surgery (Fig. 1D). This was not surprising as Lp(a) levels are determined mainly by genetic factors and remain relatively constant throughout lifetime ((27) and references therein).

Together, the results show that inflammatory markers including NEFA, TBARS, and SAA are elevated in the basal versus control LDL (Fig. 1A–C). Although NEFA showed a marginal decrease, while TBARS and SAA showed a significant decrease after surgery, they remained higher than controls. These findings, taken together with elevated plasma CRP and average BMI∼30 (borderline overweight/obese) one year after surgery, indicate continued obesity-associated inflammation.

### LDL binding to cell receptors shows a proatherogenic shift in obesity

**Fig. 2:**
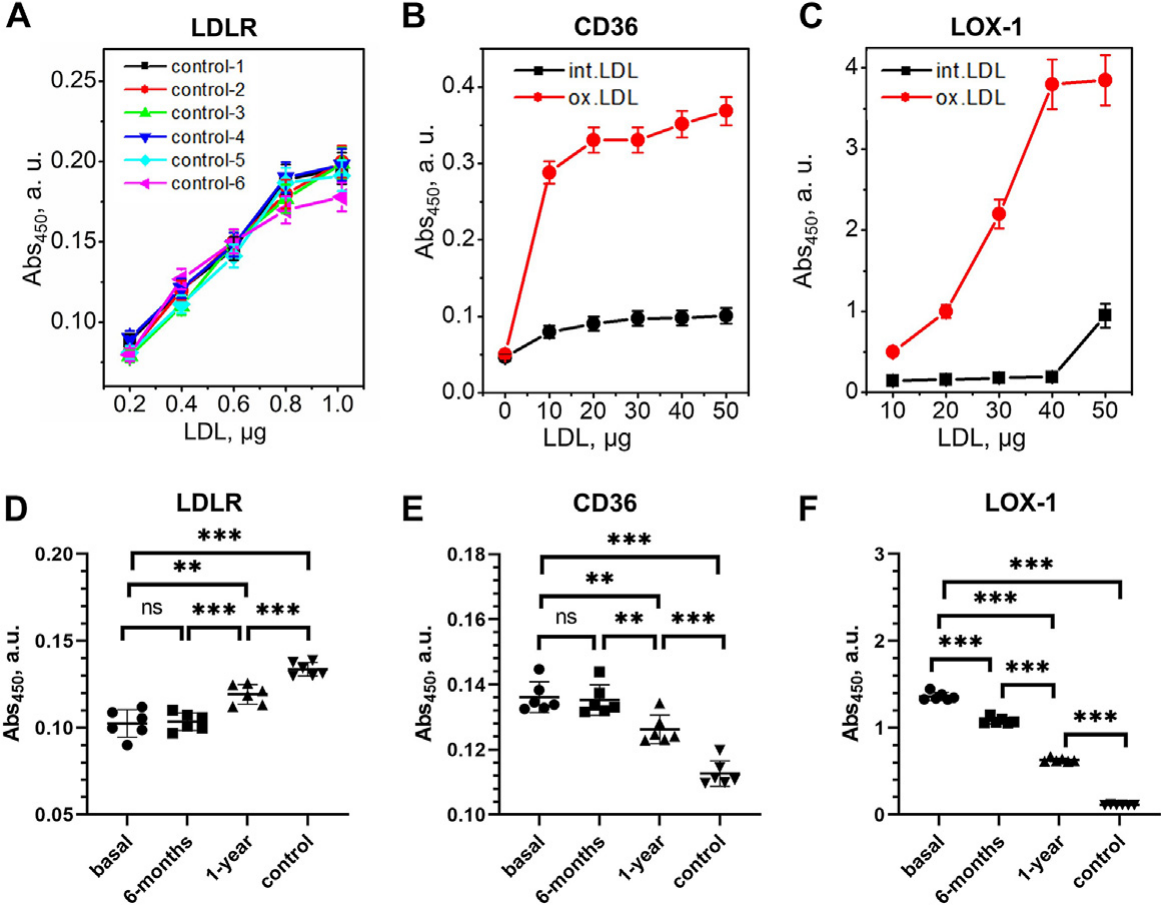
LDL binding to cell receptors measured by ELISA at pH 7.4. A–C: Validation assays for binding of control LDL to cell receptors; (A) LDLR, (B) CD36; (C) LOX-1. The receptors were immobilized on the wells and LDL binding was measured by ELISA using increasing concentration of control LDL that were either intact (for binding to LDLR) or have been oxidized by copper as described in Materials and methods (for binding to CD36 and LOX-1). The absorbance values were corrected for nonspecific binding using BSA-coated wells as a control. The binding was measured using increasing LDL concentrations at pH 7.4. D–F: Measurements of LDL binding to cell receptors; (D) LDLR, (E) CD36, (F) LOX-1. LDL protein concentration was 0.5 μg for LDLR, 5 μg for CD36, or 10 μg for LOX-1. Each data point represents an average of five independent measurements. The bars show median ±SD; ns, not significant, ∗*P* < 0.05, ∗∗*P* < 0.01, and ∗∗∗*P* < 0.001. LDLR, LDL receptor.

To explore whether obesity influences functional interactions of LDL, we analyzed LDL binding to key cell receptors involved in LDL homeostasis. LDL binding to human LDLR, CD36, and LOX-1 was quantified by ELISA. Extracellular domains of these receptors were immobilized on a 96-well plate. The assay was validated using control LDL that were either intact (for binding to LDLR) or oxidized by copper (for binding to CD36 and LOX-1) as described in Materials and methods and shown in Fig. 2A–C. Basal obese LDL showed decreased binding to LDLR compared to controls (Fig. 2D), suggesting that LDLR binding is adversely affected by altered apoB conformation, biochemical modifications of apoB, and/or increased LDL aggregation (described below). LDL binding to LDLR improved one year after surgery but remained lower than controls, suggesting that LDL functionality was partially restored.

Conversely, basal LDL showed significantly higher binding to CD36 and LOX-1 than controls (Fig. 2E, F). After surgery, the binding to CD36 and LOX-1 progressively decreased but remained higher than controls. Since CD36 and LOX-1 bind to oxidized LDL lipids, this result is in excellent agreement with partial decrease in lipid peroxidation products observed in these LDL (Fig. 1B). SAA may also contribute to the observed effect, since SAA can bind to scavenger receptors such as CD36 (42), and SAA levels in obese LDL decline after surgery but remain higher than controls (Fig. 1C).

Elevated NEFA observed in obese LDL, which persisted postoperatively (Fig. 1A), could also contribute to altered receptor binding, perhaps by altering LDL surface properties and promoting particle aggregation and fusion (31).

### LDL binding to extracellular matrix components increases in obesity

**Fig. 3:**
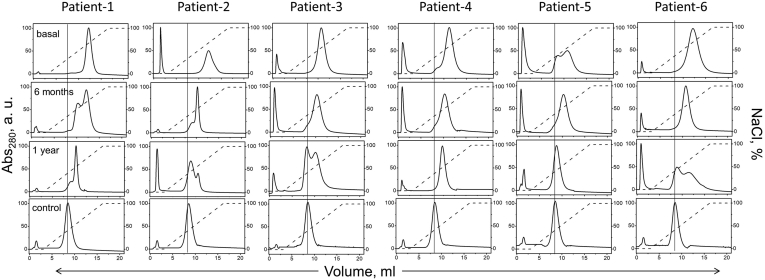
LDL binding to heparin explored using heparin affinity chromatography. Dashed line indicates NaCl gradient. Normolipidemic LDL binds heparin and elutes at 30% NaCl (control, vertical line); unbound LDL elutes at 0% (0 M) NaCl; 100% corresponds to 1 M NaCl.

In the next series of experiments, LDL binding to various extracellular matrix components was explored. First, LDL binding to heparin, a highly sulfated mimetic of heparan sulfate, was assessed by heparin affinity chromatography. LDL binding to heparin is mediated via the basic sites on apoB, some of which overlap the LDLR-binding site (19). Control LDL showed one main peak that eluted with ∼30% (or ∼0.3 M) NaCl (Fig. 3). Basal obese LDL showed a major peak shift to higher salt (elution with ∼50%, or ∼0.5 M NaCl), indicating stronger heparin binding. An additional narrow peak representing unbound LDL was observed in some samples (Fig. 3), probably reflecting glycation and oxidative cross-linking of apoB lysines in obese LDL (21). After surgery, most samples showed the major peak split into high-salt and low-salt components; the overall LDL population progressively shifted to lower salt, suggesting that heparin binding progressively declined 6 months and one year after surgery but remained higher than controls (Fig. 3).

**Fig. 4:**
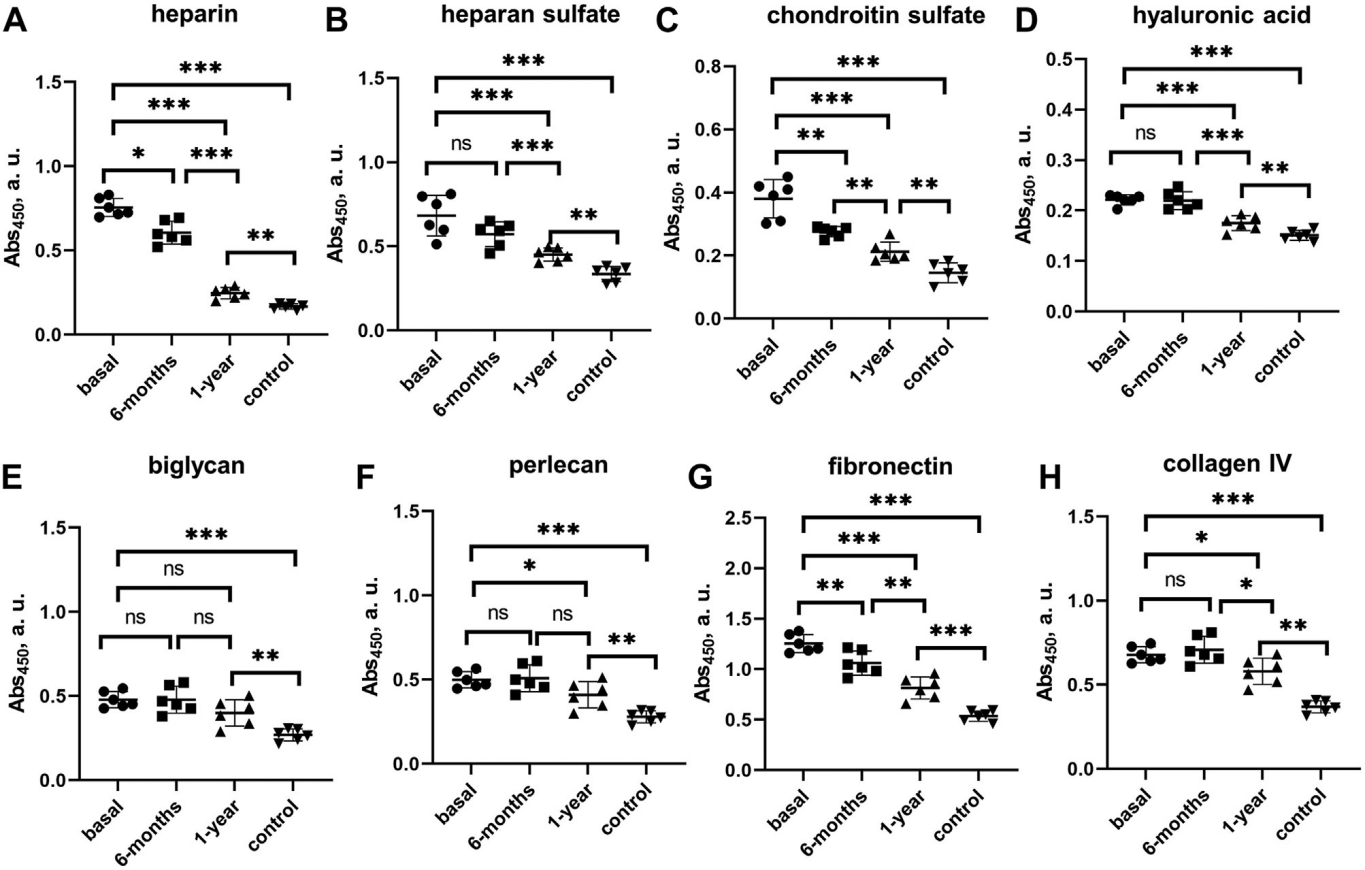
LDL binding to matrix components measured by ELISA at pH 7.4. LDL (10 μg protein) was added to the matrix immobilized on the well plate. A: heparin, (B) heparan sulfate, (C) chondroitin sulfate, (D) hyaluronic acid, (E) biglycan, (F) perlecan, (G) fibronectin, and (H) collagen-IV. Well coating with sulfated GAGs was verified by colorimetric assay as described in Materials and methods. The assay was validated using control LDL (supplemental Fig. S2). The absorbance values were corrected for nonspecific binding using BSA-coated wells as a control. Each data point represents an average of five independent measurements. The bars show median ±SD; ns, not significant, ∗*P* < 0.05, ∗∗*P* < 0.01, and ∗∗∗*P* < 0.001. GAG, glycosaminoglycan.

Next, we used ELISA to verify this observation and extend it to other matrix components, whose binding to all LDL samples was measured simultaneously. LDL can bind to various matrix components in the endothelial space (18, 43), including GAGs differing in charge and sulfation (heparan sulfate, chondroitin sulfate, hyaluronic acid), proteoglycans (biglycan, perlecan, etc.) and matrix proteins (e.g., fibronectin and collagens) (34). To quantify LDL binding to these matrix components, we immobilized them on a 96-well plate. Compared to control LDL, which bound all these components at pH 7.4, basal obese LDL showed stronger binding (Fig. 4 and supplemental Fig. S2). Heparin binding decreased 6 months postoperatively, with an even greater decrease after one year, but remained higher than controls (Fig. 4A), in excellent agreement with heparin affinity chromatography (Fig. 3). Remarkably similar trends were observed by ELISA for LDL binding to heparin, heparan sulfate, chondroitin sulfate, hyaluronic acid, biglycan, and perlecan (Fig. 4A–F). LDL binding to fibronectin and collagen generally showed similar trends: little or no changes after 6 months but decreased binding one-year postsurgery, which remained higher than controls (Fig. 4G, H). This similarity in the binding trends of LDL to diverse matrix components validated our results and suggested that they may reflect, in part, common biochemical and structural modifications of LDL in obesity.

Such modifications may potentially involve LDL aggregation and oxidation. Oxidation contributes to generation of LDL(−) that have altered apoB conformation, increased binding affinity for GAGs (44), and increased aggregation propensity (45). LDL from patients with obesity were shown to be more susceptible to enzymatically induced aggregation and oxidation but these properties normalized one year postoperatively (7). Whether the populations of aggregated LDL in plasma of patients with obesity differed from controls was unknown and was explored next.

### Aggregated LDL and electronegative LDL persist after surgery

**Fig. 5:**
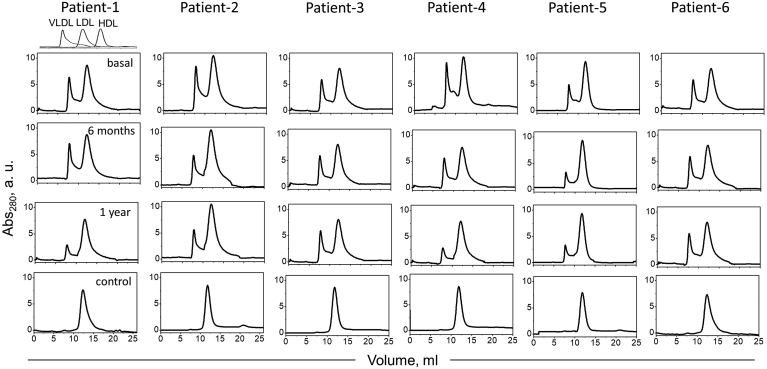
Size-exclusion chromatography profiles show particle size distribution in LDL. Profiles for single-donor LDL from six patients with severe obesity and six healthy controls are marked 1–6. Profiles of normolipidemic human HDL, LDL, and VLDL are shown for reference (top left).

Size-exclusion chromatography was used to explore the impact of obesity and bariatric surgery on the particle size distribution in nonmodified LDL (Fig. 5). Unlike control LDL, which migrated as a single peak centered at ∼12 ml, basal obese LDL showed a second peak near 7–8 ml, suggesting a subpopulation of aggregated/fused LDL particles (Fig. 5), such as those associated with diabetes (21). Since the density range used for LDL isolation (1.019–1.060 g/ml) was well above the VLDL density range (0.83–1.006 g/ml), it is unlikely that this peak was due to contamination by VLDL remnants. To verify that this peak represented aggregated/fused LDL, we explored control LDL that were either intact or have been vortexed for 10 s to avoid major biochemical modifications, followed by immediate analysis by size-exclusion chromatography as described above and by negative-stain scanning electron microscopy as previously described (26, 46). Unlike intact control LDL that showed a single peak and appeared on electron micrographs as monodisperse homogeneous particles ∼24 nm in diameter, vortexed LDL showed a second peak at ∼8 ml that represented aggregated and fused particles seen on electron micrographs (supplemental Fig. S3), supporting our interpretation. One year after surgery, the population of aggregated/fused LDL tended to decrease in most patients (Fig. 5). This result is consistent with lipolysis and oxidation that was elevated in obese LDL but decreased postoperatively (Fig. 1A, B). In fact, lipoprotein oxidation and lipolysis with generation of NEFA, which are highly fusogenic (31), drive lipoprotein aggregation and fusion (22). In conclusion, size-exclusion chromatography suggested a large subpopulation of aggregated/fused LDL in the basal obese group, which was absent from controls, with a moderate improvement one-year post-surgery.

To determine whether bariatric surgery influenced the levels of electronegative LDL, we used anion exchange chromatography to separate LDL(−) from the major less electronegative population termed LDL(+) (supplemental Fig. S4A, B). Control group showed ≥93% LDL(+) that eluted at 0.22 M NaCl and ∼5% LDL(−) that eluted at 0.6 M NaCl. Basal obese group contained ∼20% LDL(−). In agreement with our previous study (7), the levels of LDL(−) showed a progressive postoperative decline; these levels were slightly higher than in (7) and have not quite reached those of lean controls after one year, which could be due to different columns used in different laboratories (HiTrap Capto Q in the current study conducted in Boston versus MonoQ 5/50 GL in our prior work conducted in Barcelona) along with differences in the patients’ subgroup and sample storage.

In summary, populations of aggregated/fused LDL and LDL(−) increase in severe obesity and are partially normalized one-year postsurgery (Fig. 5 and supplemental Fig. S4). Lipolysis and oxidation, which are elevated in the basal and postsurgery LDL versus controls (Fig. 1A, B), probably provide a key contribution to the increased population of these proatherogenic particles.

### Probable origins of elevated NEFA in LDL but not in plasma of patients with obesity

Basal levels of plasma NEFA were not significantly different from controls (supplemental Table S1). In contrast, basal levels of LDL-bound NEFA were significantly higher than controls (Fig. 1A). This difference could result from several factors, including increased lipolytic activity of Lp-PLA_2_ in obesity (7).

Another potential factor was reduced ability of human serum albumin to bind NEFA (47). To test whether the amounts of NEFA bound by human serum albumin were altered in obesity, we isolated albumin from the lipoprotein-deficient serum using affinity chromatography and quantified albumin-bound NEFA. The results suggested a small but significant reduction in bound NEFA levels in basal versus control groups, with postoperative group showing intermediate levels (supplemental Fig. S5). Posttranslational albumin modifications such as oxidation (48) perhaps contributed to this effect.

Taken together, the results in Supplemental Table S1, Fig. 1A, supplemental Fig. S5, and (7) suggest that elevated NEFA in LDL but not in plasma of patients with obesity reflect, at least in part, increased enzymatic activity of Lp-PLA_2_, perhaps compounded by a small decrease in albumin’s ability to sequester NEFA.

### Oxidation and lipolysis increase LDL binding to matrix components

**Fig. 6:**
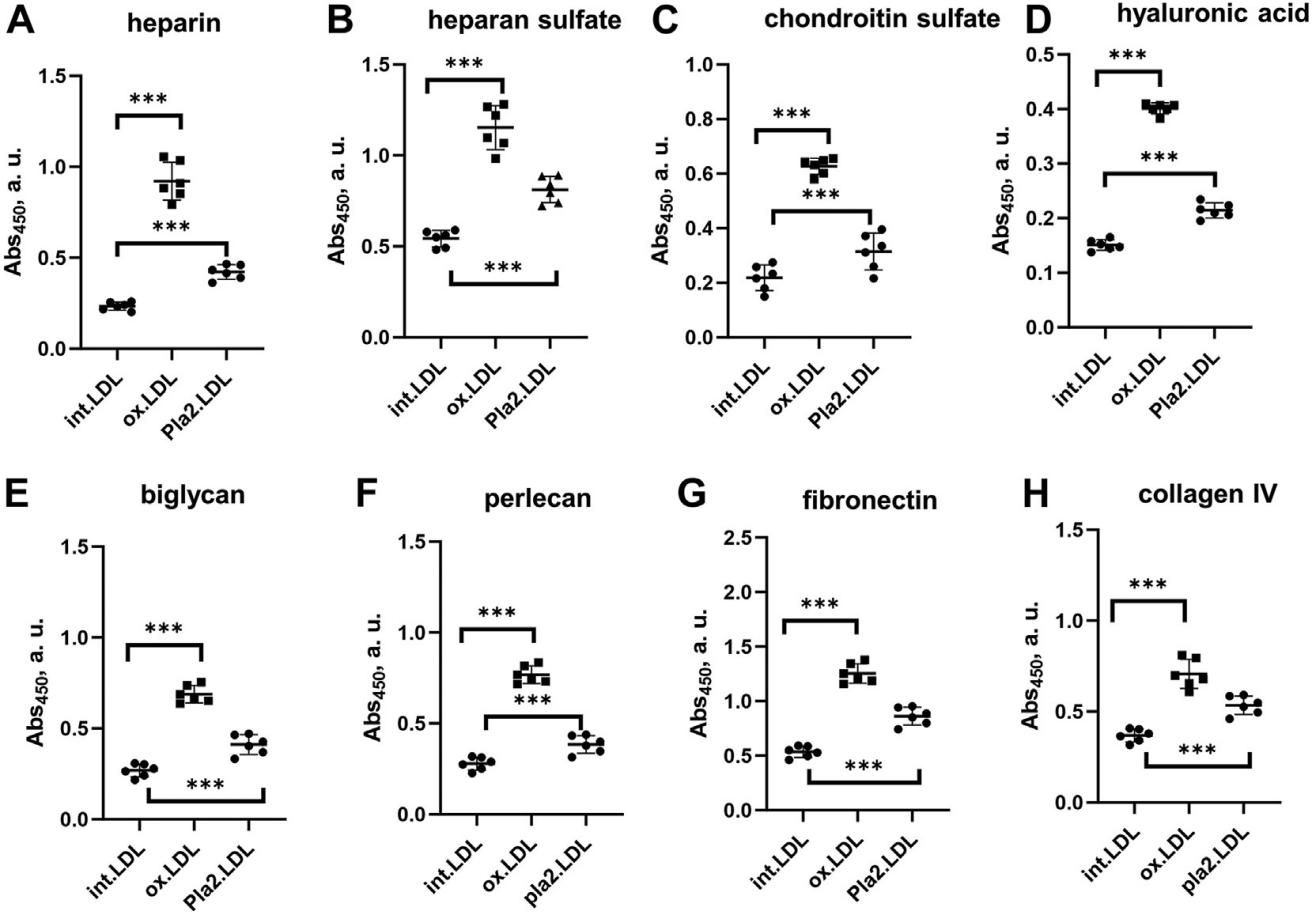
Effects of LDL oxidation and lipolysis in vitro on LDL binding to matrix components measured by ELISA at pH 7.4. Well coating with sulfated GAGs was verified by a colorimetric assay as described in Materials and methods. The absorbance values were corrected for nonspecific binding using BSA-coated wells as a control. Control LDL from lean single donors were modified in vitro by copper to generate oxidized LDL (ox.LDL) or by secretory PLA_2_ to generate PLA_2_-treated LDL (pla2.LDL) as described above in supplemental Methods. Lipolysis of PLA_2_-treated LDL was monitored by NEFA levels as shown in supplemental Fig. S6. Next, modified LDL (10 μg protein) were added to the matrix component immobilized on the well plate, and the binding was measured to (A) heparin, (B) heparan sulfate, (C) chondroitin sulfate, (D) hyaluronic acid, (E) biglycan, (F) perlecan, (G) fibronectin, and (H) collagen type-IV. Nonmodified intact LDL (int.LDL) are shown as a control. Each data point represents an average of five independent measurements. The bars show median ±SD; ns, not significant, ∗*P* < 0.05, ∗∗*P* < 0.01, and ∗∗∗*P*<0.001. GAG, glycosaminoglycan; PLA_2_, phospholipase A_2_.

To test the direct effects of lipolysis and oxidation of LDL on matrix binding in the absence of other obesity-related modifications, we used control LDL from lean subjects. These LDL were modified in vitro either by oxidation using CuSO_4_ or by lipolysis using secretory PLA_2_ as described in Materials and methods. The effects of these modifications on LDL binding to various matrix components were quantified by ELISA. The results show that both oxidation and phospholipid lipolysis increase LDL binding to all matrix components explored, with a larger increase seen in copper-oxidized LDL (Fig. 6). These results support the conclusion that oxidation and lipolysis are key contributing factors to the proatherogenic changes observed in LDL functionality in obesity.

In summary, our results revealed that LDL acquire proatherogenic properties in obesity, which are partially normalized one year after bariatric surgery. These properties include: decreased LDL binding to LDLR (Fig. 2D); increased LDL binding to CD36 and LOX-1 (Fig. 2E, F), which reflects increased lipid peroxidation, perhaps with a contribution from LDL-associated SAA (Fig. 1B, C); increased LDL binding to various matrix components (Figs. 3 and 4), which stems, at least in part, from increased LDL oxidation and lipolysis (Fig. 6); a large subpopulation of aggregated/fused LDL (Fig. 5), which probably contributes to increased matrix binding; increased population of LDL(−) (supplemental Fig. S4, (7)), which reflects elevated NEFA levels in obese LDL (Fig. 1A) and is proatherogenic. Oxidative stress and lipolysis due to obesity-associated inflammation, which is indicated by elevated levels of TBARS, NEFA, and SAA in basal and postoperative LDL (Fig. 1A–C), as well as increased plasma CRP one year postoperatively (7), contribute to these proatherogenic LDL alterations.

## Discussion

To our knowledge, current analysis is the first comprehensive study of human LDL in the context of multiple matrix interactions and the first study that indicates impaired LDL functionality in obesity and directly links it to the risk of developing CVD. The results, supported by the previous study in a larger patients’ group, show that even though obesity is not associated with elevated LDL levels, and LDL have similar content of the main lipids and proteins in obese and in healthy lean subjects (7), obesity induces biochemical and functional changes in LDL making them more proatherogenic.

Our results show that these changes in LDL result, at least in part, from obesity-induced inflammation and are partially resolved one year after bariatric surgery; they include elevated NEFA, elevated lipid peroxidation, and elevated SAA (Fig. 1A–C). Elevated oxidation and NEFA levels promote LDL aggregation/fusion and lead to increased population of LDL(−) (Fig. 5 and supplemental Fig. S4) (7), which is thought to be pro-atherogenic (12, 24, 25, 43). Furthermore, SAA binding to GAGs, particularly heparan sulfate (49), likely contributes to lipoprotein retention in the extracellular matrix, while SAA binding to scavenger receptors such as CD36 (42) may contribute to proatherogenic LDL uptake by arterial macrophages.

These obesity-associated changes adversely influence essential aspects of LDL functionality: *1*) they diminish nonatherogenic binding to LDLR (Fig. 2D); *2*) they enhance proatherogenic binding to CD36 and LOX-1 receptors (Fig. 2E, F); *3*) they enhance proatherogenic binding and retention by extracellular matrix components (Fig. 4). Enhanced LDL binding to CD36 and LOX-1 probably results from increased lipid oxidation in obesity (Fig. 1B), which is consistent with reduced antioxidant capacity of HDL observed in these patients (7). Enhanced LDL binding to various matrix components, which is mediated mainly by apoB (34), probably reflects combined effects of LDL lipolysis and oxidation, as suggested by our experiments using control LDL modified in vitro in the absence of other changes (Fig. 6). LDL aggregation/fusion may contribute to enhanced binding to matrix components by creating multivalent ligands for such binding (25). Elevated SAA in obese LDL (Fig. 1C) may also contribute to their stronger binding to scavenger receptors (42), GAGs (50), and matrix proteins (51). Although these harmful changes in obese LDL substantially diminish one year postoperatively, they do not reach the levels of healthy lean controls (Fig. 1, Fig. 2, Fig. 3, Fig. 4, Fig. 5 and supplemental Fig. S4). The patients’ BMIs also remain higher than controls (supplemental Table S1).

These proatherogenic changes in obese LDL directly link the obesity-associated inflammation with LDL functionality and the risk of CVD. Moreover, they provide new insights into benefits and limitations of bariatric surgery in normalizing lipoprotein metabolism, in particular LDL functionality. Such insights may help better understand and, ultimately, manage the risk of CVD in patients with obesity.

Elevated NEFA are a hallmark of several pathological processes, including diabetes, metabolic syndrome, and obesity (52). Increased levels and activity of Lp-PLA_2_ were observed in apoB-containing lipoproteins from patients with obesity (7). Additionally, levels and activity of secretory PLA_2_ are elevated in inflammation (47, 53). The combined effects perhaps contribute to elevated NEFA observed in basal versus control LDL in the current study (Fig. 1A). Notably, total NEFA did not significantly differ between basal and control plasma (7), suggesting a mechanism contributing to elevated NEFA in LDL but not in plasma of individuals with obesity. In healthy state, most circulating NEFA are cleared by albumin, yet during inflammation albumin’s ability to sequester NEFA decreases; as a result, bound NEFA shift to LDL (supplemental Fig. S5 and (47)). Therefore, elevated NEFA in basal LDL can be attributed, at least in part, to inflammation associated with obesity. One year after the bariatric surgery, the patients lost weight but have not reached the lean status (supplemental Table S1). Therefore, the obesity-associated inflammation was diminished but not entirely abolished, and hence, NEFA levels in postoperative LDL remained higher than in controls (Fig. 1A). Since oxidation promotes LDL(−) formation (24) and NEFA promote lipoprotein aggregation and fusion (31), we propose that elevated levels of oxidation and NEFA contribute to increased LDL(−) and to LDL aggregation which, in turn, adversely affects receptor and matrix binding by LDL.

Obesity-associated inflammation emerges as the key contributing factor to altered LDL functionality in obesity, as summarized in supplemental Fig. S7. Inflammation may have a triple effect on LDL in obesity: it enhances the expression of Lp-PLA_2_ and secretory PLA_2_; it decreases the albumin’s ability to sequester NEFA; and it increases the oxidative stress. Since Lp-PLA_2_ degrades only oxidized phospholipids, minimal oxidation is needed to activate this LDL-associated lipase. Due to this triple action, LDL from subjects with obesity have elevated electronegative and aggregated LDL. Additionally, the levels of small dense LDL, an aggregation-prone proatherogenic LDL subclass, are also increased in obesity (7). In fact, the presence of small dense LDL in basal obese samples (7) could underlie some of the atherogenic properties reported in the present study, since it is known that small dense LDL has a poor affinity for the LDL receptor (54, 55) and also increased binding to matrix components (55, 56). In addition, small dense LDL is highly susceptible to oxidation (57), which could account for increased LDL(−) population, increased TBARS content, and increased interaction with CD36 and LOX-1 displayed by LDL from basal obese patients. These properties, together with decreased LDL affinity for LDLR and increased affinity for scavenger receptors and matrix components revealed in the current study, confer pro-atherogenic properties to obese LDL.

These findings compel us to propose that proatherogenic LDL alterations are central to the accelerated CVD development in obesity. Weight loss following bariatric surgery can partially normalize these LDL alterations by reducing the systemic obesity-associated inflammation.

### Study limitations and future work

The small sample size is the major limitation of the current study. LDL oxidation during long data collections cannot be excluded; however, all plasma samples had a similar storage time at −80°C, and the behavior of control and obese samples was clearly different. Furthermore, the results have been validated by consistent trends observed across different types of experiments. Future studies with larger patients’ groups and longer follow-up periods will help expand our findings and determine whether the beneficial postoperative trends continue beyond one year and follow the weight loss trends. Future studies should also test our hypothesis that current findings are not limited to severe obesity but can be extrapolated to obesity. Additionally, the effects of other comorbidities on LDL functionality should be considered, including diabetes, hyperlipidemia, smoking, high blood pressure, and genetic traits. Future studies should also consider whether obesity-induced alterations in cell receptors and matrix components influence functional LDL interactions.

## Data availability

The data are available from the corresponding authors upon reasonable request.

## Ethics approval and consent to participate

The study was conducted according to the guidelines of the Declaration of Helsinki upon approval by the Ethics Committee of the Hospital de la Santa Creu I Sant Pau (protocol code IIBSP-APO-2013-105, approved 01-21-2014). Written informed consent was obtained from all subjects prior to their involvement in the study.

## Consent for publication

All authors give their consent to publish this study.

## Supplemental data

This study contains supplemental information.

## Conflict of interest

The authors declare that they have no conflicts of interest with the contents of this article.
