# Supplementary material for: LDL binding to cell receptors and extracellular matrix is proatherogenic in obesity but improves after bariatric surgery
Source: J Lipid Res. 2023 Sep 28;64(11):100451. doi: 10.1016/j.jlr.2023.100451 (PMC10665669; doi:10.1016/j.jlr.2023.100451)
Supplement: Supplemental data [file mmc1.pdf]

LDL binding to cell receptors and extracellular matrix is proatherogenic in obesity but improves after bariatric surgery

Shobini Jayaraman, Antonio Pérez, Inka Miñambres, Jose Luis Sánchez-Quesada, Olga Gursky

SUPPLEMENT

CONTENT:

1. Supplemental Table S1
2. Supplemental Figures S1-S7
3. Resources Table
4. Supplemental References

Supplemental Table S1. Anthropometric and clinical characteristics (in black) and lipid levels in plasma (blue) and in major plasma lipoproteins (red) of patients with obesity and lean healthy controls whose LDL were used in the current study.

Parameters (units)	Obese (n = 6)			Control group (n = 6)
Age (years)	46.3 ± 12.9			38.7 ± 14.6
Gender (M/F)	2/4			2/4
	Basal	6 months	1 year	
BMI (kg/m^2)	40.9 ± 3.5	33.7 ± 3.3 [a]	29.5 ± 3.2 [a,b]	22.8 ± 2.4 [a,b,c]
HbA1c (%)	5.86 ± 0.77	5.41 ± 0.30	5.54 ± 0.66	5.09 ± 0.17
Cholesterol (mmol/l)	4.73 ± 0.61	4.10 ± 0.97 [a]	4.98 ± 1.31 [b]	4.58 ± 0.67 [b]
Triglycerides (mmol/l)	1.67 ± 0.92	1.12 ± 0.21 [a]	1.06 ± 0.30 [a]	0.73 ± 0.16 [a,b,c]
ApoB (g/l)	1.00 ± 0.20	0.84 ± 0.19 [a]	1.01 ± 0.21 [b]	0.81 ± 0.12 [a,b]
NEFA (mmol/l)	0.58 ± 0.19	0.57 ± 0.31	0.60 ± 0.22	0.54 ± 0.14
hsCRP (mg/L)	10.48 ± 6.18	3.79 ± 3.39 [a]	3.28 ± 2.15 [a]	0.90 ± 0.32 [a,b,c]
VLDL-Chol (mmol/l)	0.70 ± 0.26	0.52 ± 0.10 [a]	0.49 ± 0.14 [a]	0.34 ± 0.07 [a]
LDL-Chol (mmol/l)	2.92 ± 0.49	2.57 ± 0.92 [a]	3.49 ± 1.08 [b]	2.47 ± 0.13 [b]
HDL-Chol (mmol/l)	1.11 ± 0.24	1.01 ± 0.16	1.34 ± 0.27 [a,b]	1.77 ± 0.52 [a,b]

[a] $p < 0.05$ vs. basal.
[b] $p < 0.05$ vs. 6 months.
[c] $p < 0.05$ vs. 1 year.
BMI: Body mass index.

SUPPLEMENTAL FIGURES

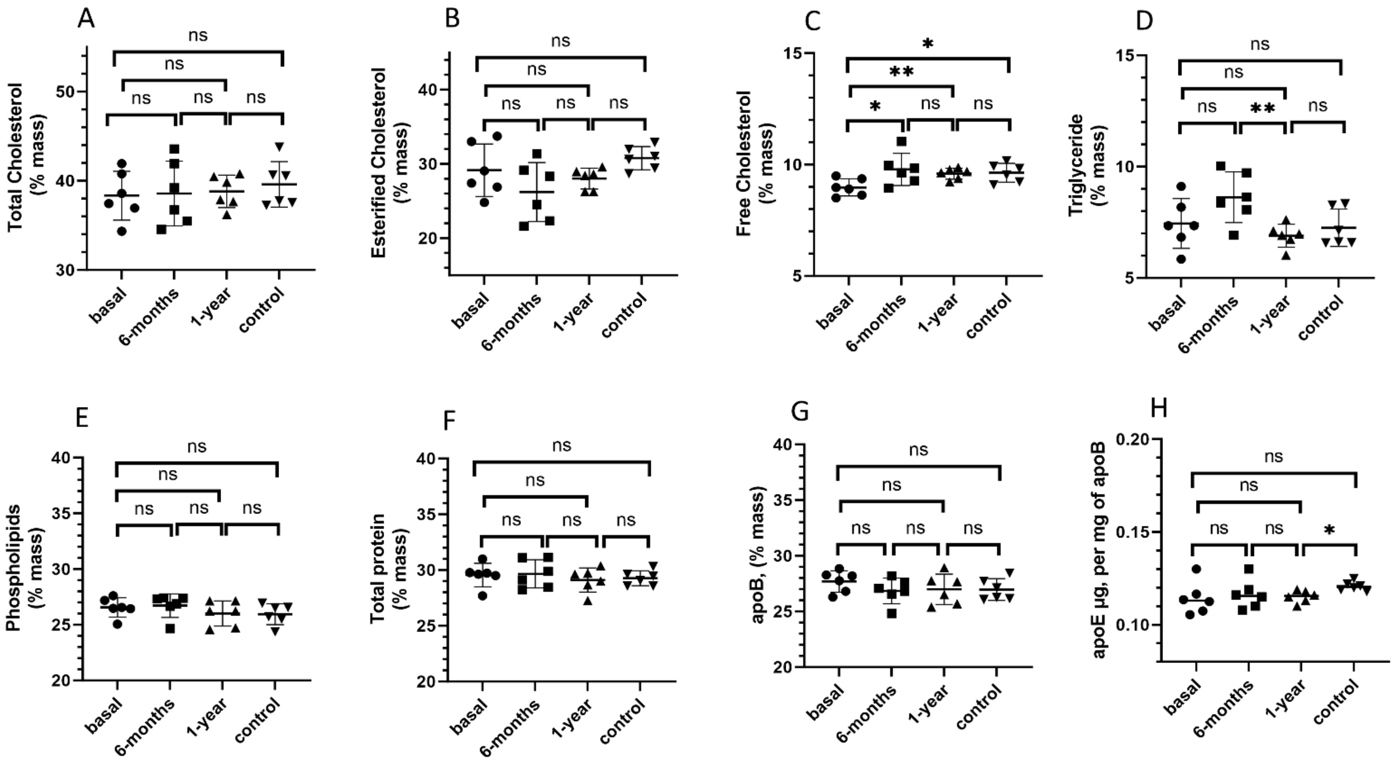

Supplemental Fig. S1. Biochemical composition of single-donor LDL used in the current study. LDL lipids were quantified using commercially available kits. A: total cholesterol, B: esterified cholesterol, C: free (non-esterified) cholesterol, D: triglycerides, E: phospholipids. Total protein was estimated using the Bradford assay (F). ApoB (G) and apoE levels (H) were quantified by ELISA. Data are calculated from percent mass of each component. Each data point represents an average of five independent measurements. The bars show median ±STD; ns – not significant, * p<0.05, ** p 0.01, *** p<0.001.

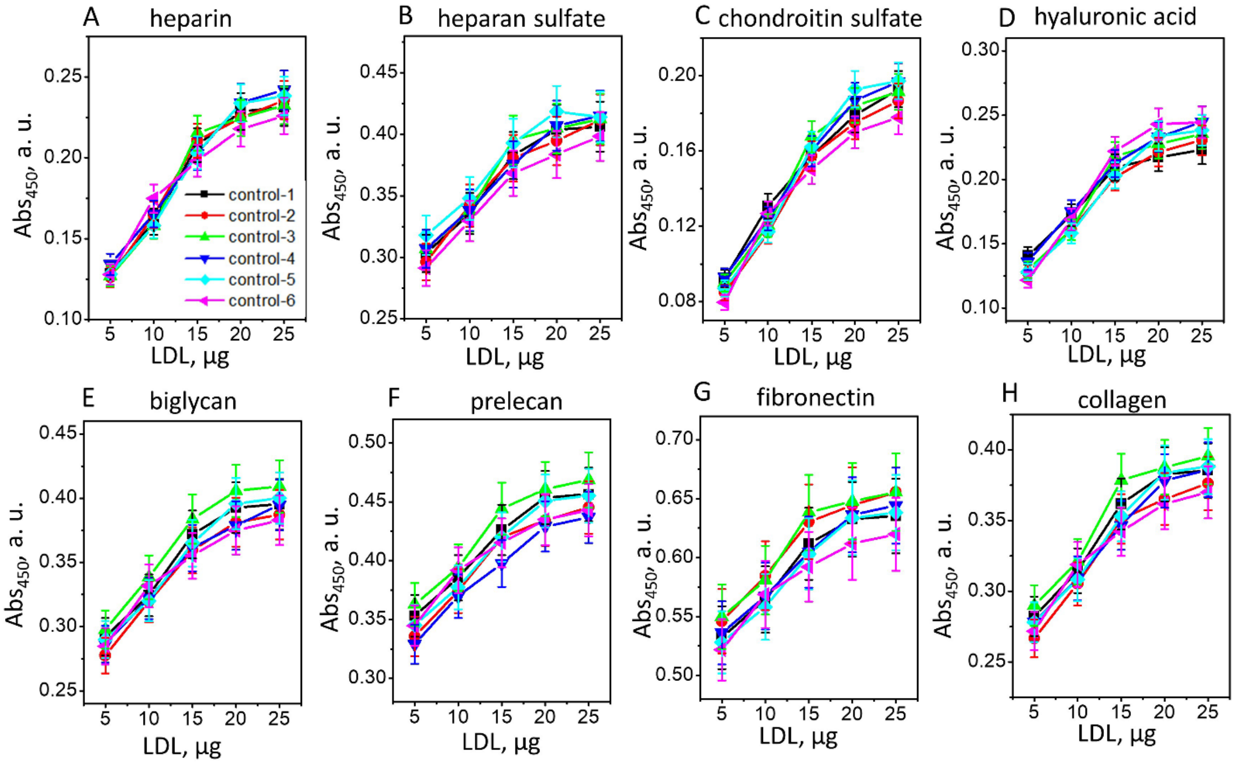

Supplemental Fig. S2. Dose-dependent biding of LDL to various extracellular matrix components. The components are indicated in the panels; A: heparin, B: heparan sulfate, C: chondroitin sulfate, D: hyaluronic acid, E: biglycan, F: perlecan, G: fibronectin, and H: collagen-IV. These components were immobilized in the wells and the binding was measured by ELISA at pH 7.4 using increasing amounts of LDL (from 5 to 25 μg protein) as described in Methods. The absorbance values were corrected for non-specific binding using BSA-coated wells as a control. Each data point represents an average of five independent measurements. The bars show ±STD. Data using single-donor LDL from individual healthy controls are color-coded.

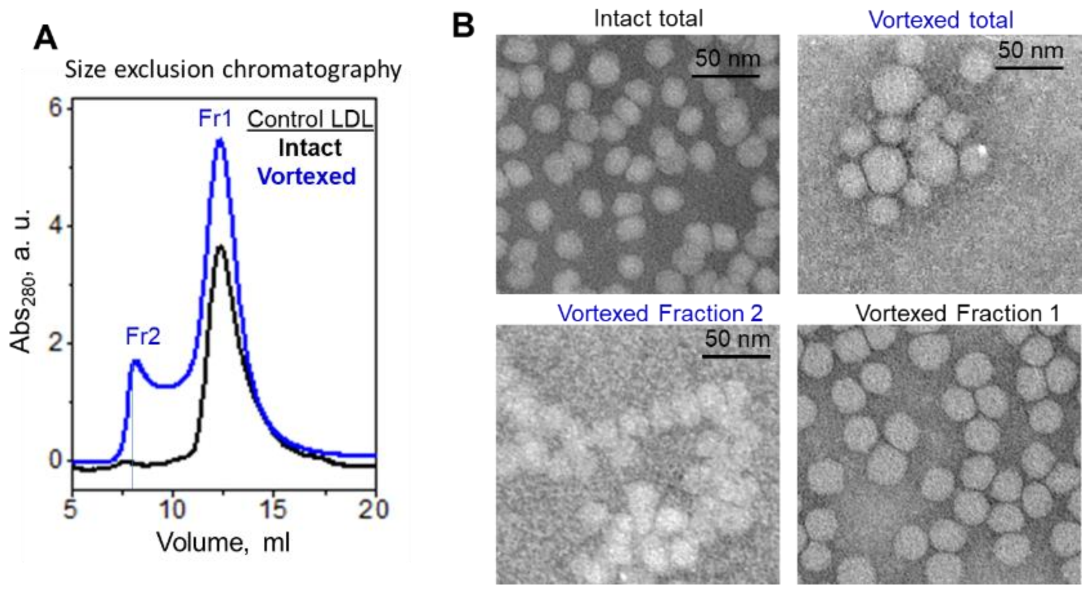

Supplemental Fig. S3. Verification of the origin of the second peak in size exclusion chromatography of LDL. Control single-donor LDL was either intact (black) or has been vortexed for 10 sec (blue), followed by analysis using size-exclusion chromatography. Two peaks were separated and, together with total LDL (intact and vortexed), were visualized using negative-stain transmission electron microscopy as previously described (1). Only vortexed LDLs, both total and peak 2 fraction that elutes at ~8 ml, show aggregated and fused particles.

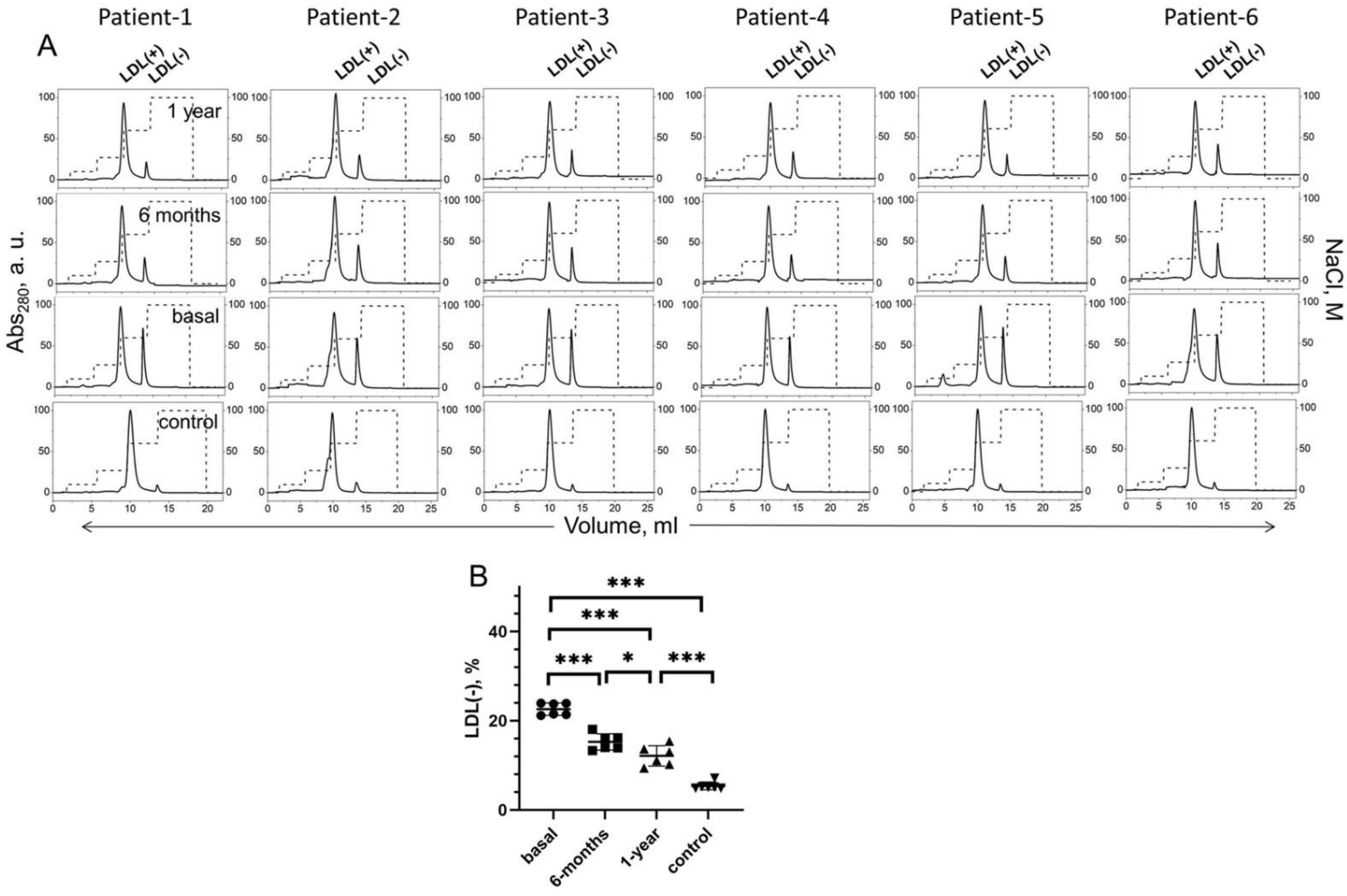

Figure S4. Anion exchange chromatography to assess LDL(-) and LDL(+) populations. A: Chromatography profiles show the major LDL subclass, LDL(+), that elutes at 0.23 M NaCl; the minor LDL(-) subclass elutes at 0.6 M NaCl. B: Quantification of LDL(-) population in the chromatography profiles. LDL(-) was quantified by peak integration using the Unicorn 5.20 workstation in the ÄKTA system using the default option. Each data point represents an average of five independent measurements. The bars show median ±STD; *p<0.05, **p<0.01, ***p<0.001.

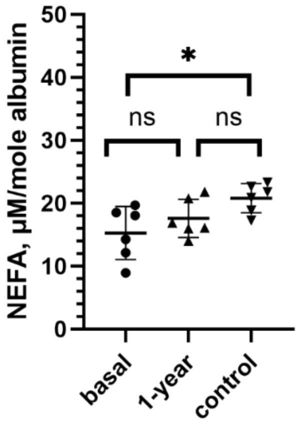

Supplemental Fig. S5. Albumin-bound non-esterified fatty acid (NEFA) levels in serum of patients with obesity before and one year after bariatric surgery compared to lean healthy controls. Human serum albumin was isolated from lipoprotein-deficient serum using albumin affinity HiTrap blue HP column as described above in supplemental Methods. Fractions were pooled between 8 and 25 ml and dialyzed against 10 mM PBS before further analysis. Endogenous NEFA levels in albumin were measured using an assay kit. Each data point represents an average of five independent measurements. The bars show median ±STD; ns – not significant, *p<0.05.

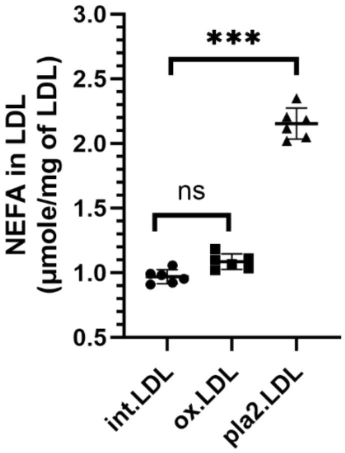

Supplemental Fig. S6. NEFA levels in modified LDL. Control LDL from six healthy lean subjects were modified by copper oxidation or by hydrolysis using secretory PLA_2 as described above in Methods and in supplemental Fig. 4 legend. NEFA levels in intact, oxidized, and in PLA_2-treated LDL were quantified as previously described (2)). Significant increase in NEFA was observed in PLA_2-treated LDL, indicating that these particles have undergone lipolysis. No significant increase in NEFA was detected in oxidized LDL. Each data point represents an average of five independent measurements; the bars show median ±STD; ns – not significant, * ***p<0.001.

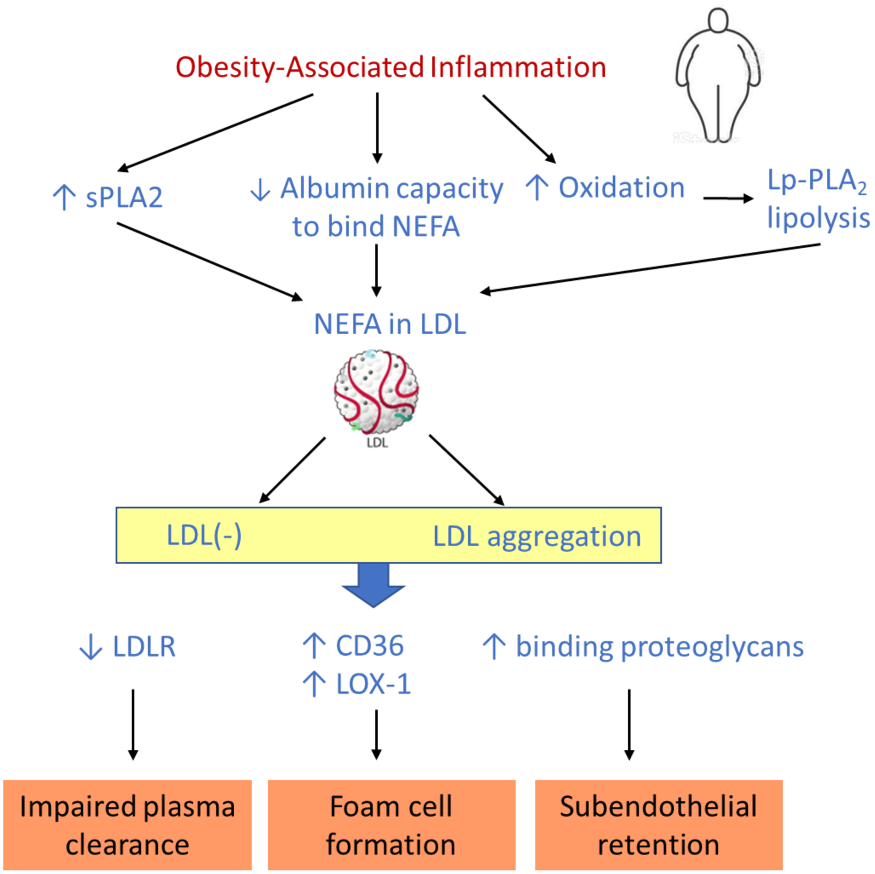

Supplemental Fig. S7. Scheme illustrating impaired LDL functionality in obesity and its putative role in the accelerated development of atherosclerosis. Inflammation has a triple effect: i) enhancing the expression of secretory PLA_2 ($sPLA_2$), ii) decreasing the albumin's ability to sequester NEFA, and iii) increasing the oxidative stress that can activate lipoprotein-associated PLA_2 ($Lp\text{-}PLA_2$) in LDL. As a result, the content of NEFA increases in LDL from obese subjects. NEFA content in LDL is the main determinant of LDL(-) and a key driver of LDL aggregation and fusion. Aggregated LDL and LDL(-) have decreased affinity for the non-atherogenic LDLR. However, these LDL readily bind the pro-atherogenic scavenger receptors CD36 and LOX-1, ultimately leading to the foam formation in the arterial wall. Aggregated LDL and LDL(-) also have increased affinity for proteoglycans and matrix proteins, thereby promoting LDL retention in the sub-endothelial space. Small dense LDL, a pro-atherogenic LDL subclass that is increased in obesity, probably contributes to these effects. As a result, LDL from patients with obesity likely play a key role in the development of atherosclerosis.

RESOURCES TABLE

Antibodies

Target antigen	Source	Catalog #
Human apoB	Santacruz	Sc-13538-HRP

Assays

Description	Source	Catalog #
Phospholipid	Fisher Scientific	EPLP-100
Triglyceride	Fisher Scientific	ETGA-200
Free fatty acid	Fisher Scientific	EFFA-100
Cholesterol	Sigma	MAK-043
Quick Start Bradford protein assay kit	Biorad	5000201

ELISA kits

Target antigen	Source	Catalog #
Human apoB	Abcam	AB190806
Human apoE	Abcam	AB233623
Human Lp(a)	Abcam	AB212165
Human SAA	Abcam	AB100635

Materials sources

No	Chemical	Vendor	Catalog #
1	Thiobarbituric acid	Sigma	T5500
2	Malondialdehyde	Sigma	36357
3	Bovine serum albumin	Sigma	A3294
4	Heparin	Sigma	H4784
5	Heparan sulfate	Sigma	H7640
6	Chlondotrin sulfate	Sigma	40583
7	Hyaluronic acid sodium salt	Sigma	840101C
8	Collagen type-I from rat tail	Sigma	C3867
9	Collagen type-IV from human placenta	Sigma	CC076
10	Recombinant human biglycan protein	Biotechne R&D	2667-CM-050
11	Fibronectin	Sigma	11051407001
12	Recombinant human endorepellin perlecan protein	Biotechne R&D	12364-ER-050
13	Tetramentylbenzidine	Fisher Scientific	34028
14	Recombinant human LDL receptor	Abclonal	RP00152
15	His-tagged extracellular ligand-binding domain of human CD36	Sino Biologicals Inc.	10752-1708H
16	Recombinant human LOX-1/OLR1 protein	Biotechne R&D	1798-LX-050
17	Phospholipase A_2 from honeybee venom	Sigma	P9279

References

1. Rull, A., S. Jayaraman, D. L. Gantz, A. Rivas-Urbina, M. Pérez-Cuellar, J. Ordóñez-Llanos, J. L. Sánchez-Quesada, and O. Gursky. 2016. Thermal stability of human plasma electronegative low-density lipoprotein: A paradoxical behavior of low-density lipoprotein aggregation. *Biochim Biophys Acta*. **1861**: 1015–1024.
2. Jayaraman, S., O. R. Chavez, A. Pérez, I. Miñambres, J. L. Sánchez-Quesada, and O. Gursky. 2020. Binding to heparin triggers deleterious structural and biochemical changes in human low-density lipoprotein, which are amplified in hyperglycemia. *Biochim Biophys Acta Mol Cell Biol Lipids*. **1865**: 158712.
